# Respiratory function modulated during execution, observation, and imagination of walking via SII

**DOI:** 10.1038/s41598-021-03147-5

**Published:** 2021-12-09

**Authors:** Antonello Pellicano, Gianluca Mingoia, Christoph Ritter, Giovanni Buccino, Ferdinand Binkofski

**Affiliations:** 1 Division for Clinical and Cognitive Sciences, Medical Faculty, RWTH Aachen University, Pauwelsstr. 17, 52074 Aachen, Germany; 2 RWTH Aachen University, Aachen, Germany; 3 Brain Imaging Facility, Interdisciplinary Center for Clinical Research, RWTH Aachen University, Aachen, Germany; 4 Division of Neuroscience, San Raffaele Scientific Institute, Faculty of Medicine, University San Raffaele, Milan, Italy; 5 Institute for Neuroscience and Medicine (INM-4), Research Center Jülich GmbH, Jülich, Germany; 6 Jülich-Aachen-Research-Alliance (JARA), Jülich, Germany

**Keywords:** Neural circuits, Sensorimotor processing, Cognitive neuroscience

## Abstract

The Mirror Neurons System (MNS) consists of brain areas active during actions execution, as well as observation-imagination of the same actions. MNS represents a potential mechanism by which we understand other's action goals. We investigated MNS activation for legs actions, and its interaction with the autonomic nervous system. We performed a physiological and fMRI investigation on the common neural structures recruited during the execution, observation, and imagination of walking, and their effects on respiratory activity. Bilateral SMA were activated by all three tasks, suggesting that these areas are responsible for the core of the MNS effect for walking. Moreover, we observed in bilateral parietal opercula (OP1, secondary somatosensory cortex-SII) evidence of an MNS subtending walking execution-observation-imagination that also modulated the respiratory function. We suggest that SII, in modulating the vegetative response during motor activity but also during observation-imagination, consists of a re-enacting function which facilitates the understanding of motor actions.

## Introduction

Action execution, observation, and imagination are fundamental processes for social interactions in humans. A *mental simulation theory* was proposed by Jeannerod^1^ which assumed that actual motor execution, action observation, and imagination are to some extent functionally equivalent as they are based on common motor representations. Indeed, there is increasing evidence that they share common neural substrates (for a review see Hardwick et al.^2^). A brain mechanism would be able to match an observed action, or an imagined one, with the motor representation employed in the actual execution of that action. The cortical areas responsible for this mechanism are known as the *mirror neuron system* (MNS)^3,4^. Mirror Neurons are a specialized subset of visuomotor neurons, originally discovered in area F5 of the monkey premotor cortex. They have the peculiarity to discharge both when the monkey performs a given action, and when he observes the same action performed by someone else^5,6^. Evidence of an MNS has been subsequently provided for other areas in the human and non-human motor pathway that largely consist in primary and premotor cortices, inferior frontal gyrus, and parietal regions^7–10^. Thus, the MNS represents a unifying mechanism active whenever motor representations are recalled as during action observation, motor imagery, dreams with a motor content and so on, even in the absence of overt action.

Walking is a complex motor behaviour with special relevance in the development of interpersonal and social interactions (Pavlova^11^, for a review). By observing walking, people can understand intentions and emotional states of the agent, even from sketchy body segments as it occurs with point-light biological motion stimuli (Johansson et al.^12^, for a review). Most of the studies assessed the cortical representation of human walking related to execution, observation, and imagery, but taken separately. Neuroimaging studies have found that the motor action of walking is associated to the activations of several cortical areas such as medial part of primary sensory-motor cortex (pSM), supplementary motor area (SMA), and premotor cortex (PM); as well as subcortical structures: basal ganglia and cerebellar vermis^13–15^ more rarely the involvement of occipital and associative temporoparietal cortices was observed. Similar brain activity was also observed during the pure motor imagery of walking^16–19^. In addition to this, a pattern of parietal, frontal and temporo-occipital activations which is compatible with brain function necessary to perform walking execution was also displayed during the observation of walking^20,21^.

In other studies, the combination of walking execution and imagery was investigated^18,22^. Apart from a differential activation in the primary motor cortex, which was engaged only during actual execution, a pattern of activation was largely shared by both the tasks. Motor imagery of walking has been also compared to walking observation^23^. Among common cortical structures subserving both tasks, there were dorsal PM area bilaterally, left SMA, and right superior parietal lobule (SPL). In summary, results indicate recruitment of the cortical sensory-motor system, with a significant convergence between execution and imagery on the one hand and imagery and observation on the other. Indeed, the notion that motor imagery and motor execution share common neural substrates is also well established for hand actions^24–29^. To note, since walking movements are rather hard to be performed in a scanner, in the mentioned studies where PET was employed, walking was executed offline and before the scanning session. In the fMRI studies, instead, participants performed a walking imagery task since imagery and actual walking execution partially share the same neural substrates. To the best of our knowledge, there is only one study^29^ that overcame such technical limitations by allowing participants to walk on a rolling cylinder while lying in the MRI scanner. This study provided evidence of common activations for walking observation (observed video clip) and actual execution of a walking movement in the scanner, in the bilateral dorsal premotor/supplementary motor areas and in the posterior parietal lobe.

As a whole, a unified fMRI investigation of execution, observation, and imagination for walking has not been provided yet. The first aim of the present study was to investigate the common neural structures of the central nervous system (CNS) recruited during the execution, observation, and imagination of walking, and to systematically assess the existence of a matching system for these three processes.

In the present study, physiological measures of the Autonomous Nervous System (ANS) activity, namely respiratory rate (RR) and respiration rate variability (RRV), were also employed during the acquisition of fMRI data. Some pivotal studies have shown a modulation of autonomic responses during the imagination^30–33^, as well as the observation of actions^34–36^. However these studies investigated autonomic responses at behavioral level only, and separately for observation and imagination. Moreover to the best of our knowledge, there are no studies on the brain correlates of autonomic responses found during these tasks.

Also for what concerns investigations on the ANS, physiological correlates of execution, observation, and imagination of walking actions have not been yet conducted within a unified approach. Importantly, unlike grasping or pointing, a motor activity like walking can produce larger autonomic responses which allow to better discriminate between the effects of actual movement and resting, control conditions. The second aim of the present study was to investigate the presence of autonomic markers of action observation and imagination. ANS responses (Respiration Rate, and Respiration Rate Variability) were expected to be proportional to the intensity of the observed/imagined movements: ANS responses should result stronger when a walking action is observed or imagined compared to when a low-intensity control action is given^37^. Crucially, we investigated the brain neural structures potentially involved in this modulation; thus, we explored the hypothetical anatomo-functional link between the Mirror Neurons circuits, in the CNS, and the related responses of the ANS. We suggest that the secondary somatosensory cortex (SII) plays a role in the reenactment of changes in breath frequency, consistent to perceived/imagined changes in motor efforts, as part of a neural circuit that expands the boundaries of the so far known MNS.

## Results

**Figure 1:**
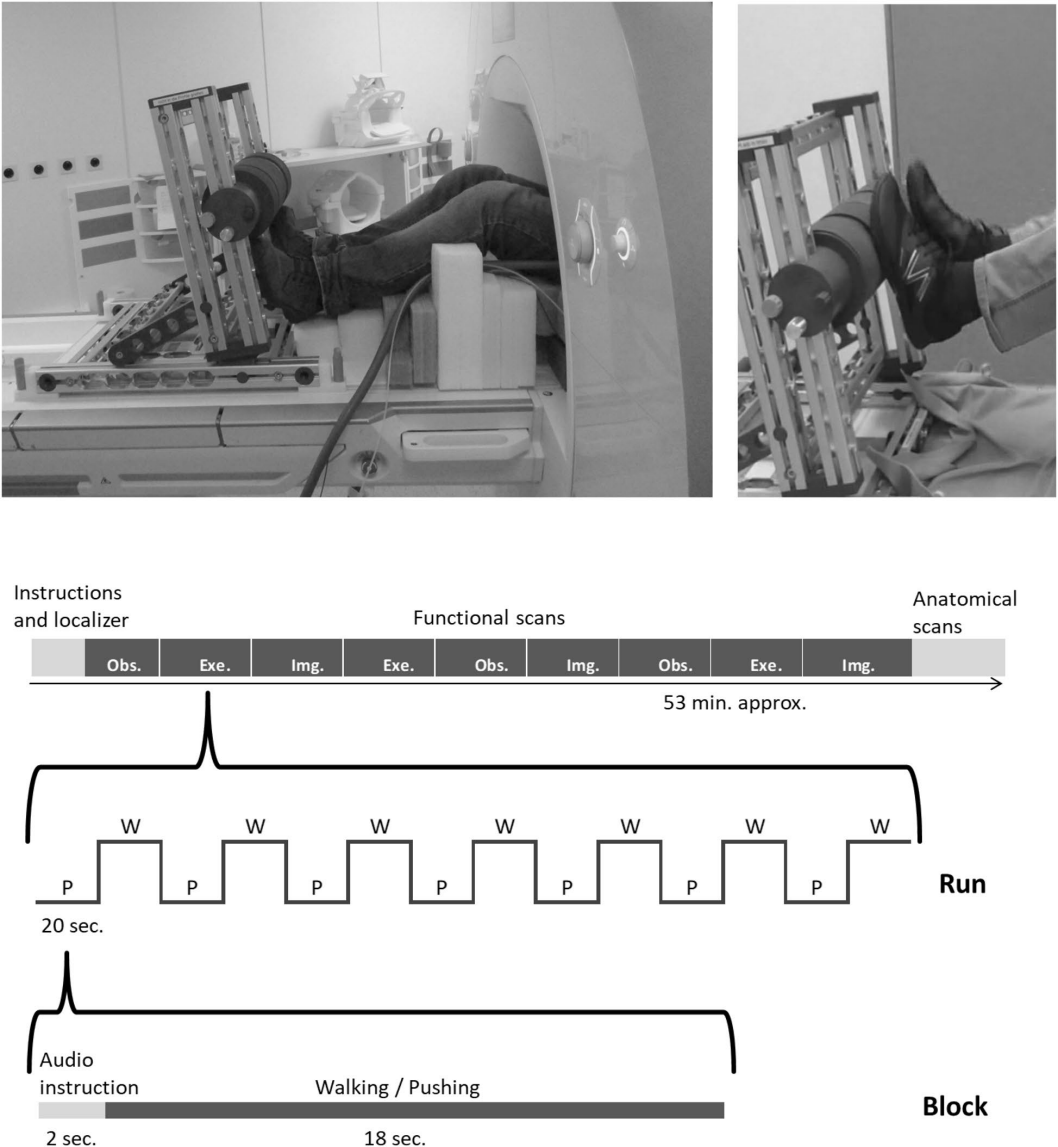
Experimental apparatus and design. The execution, observation, and imagination conditions were manipulated within-participants. Each condition consisted of the walking (“W”) and pushing (“P”) tasks that were alternated within one run. For each condition, 3 runs were given. The sequence of a total of 9 runs was pseudo-randomized between the participants.

While laying in the scanner, participants performed three tasks: execution, observation, and imagination of a walking movement; together with corresponding baselines (i.e., execution, observation, and imagination of a gentle pushing movement) (Fig. 1). Mean respiration rate (RR) and respiration rate variability (RRV) were collected for each participant.

### fMRI data results

Table 1 shows the list of the MNI standard brain coordinates of the local maxima of BOLD-signal increases as revealed by the contrast analyses between the tasks and the related baselines: walking execution—pushing execution, walking observation—pushing observation, walking imagination—pushing imagination. For simplicity, resulting contrast images will be named as *Execution*, *Observation*, and *Imagination*, respectively.

**Table 1:** MNI standard brain coordinates of the local maxima of BOLD-signal increases from the contrast analyses: walking—pushing, walking observation—pushing observation, walking imagination—pushing imagination.

Cluster Index	Anatomical region	Z	x	y	z	x	y	z
**Execution**								
1	cingulate gyrus*	3.4				18	−26	30
2	Cerebellar vermis (3) lobule I IV (prob. 100%)	4.78				2	−44	−10
	Cerebellum lobule V (prob. 56%), I IV (16%)	3.95				18	−34	−22
3	Paracentral lobule, area 4a (prob. 15%)	6.06				10	−32	62
	Paracentral lobule, area 4a (prob. 65%)	5.59	−6	−28	60			
	Paracentral lobule, area 4a (prob. 39%), area 5 M (SPL, 18%), area 3a (6%)	5.55	−8	−38	68			
	Paracentral lobule, area 4a (prob. 12%), area 3a (6%)	5.51	−10	−38	72			
**Observation**								
1	Putamen	4.03	−20	0	8			
2	Middle frontal gyrus*	3.94				44	−4	62
	Precentral gyrus*	3.82				48	−6	60
	Middle cingulum, BA 24*	3.46				14	−20	42
3	Thalamus parietal (prob. 47%), somatosensory (22%), temporal (8%)	4.84	−12	−26	−4			
	Thalamus parietal (prob. 22%), somatosensory (18%), Premotor (12%)	3.64				10	−24	−4
4	Inferior parietal lobule*	5.8	−42	−32	26			
	Superior parietal lobule, area 5L (SPL, 67%), area 2 (34%)	5.77	−26	−44	52			
	Precentral gyrus (BA 44 prob. 18%)	4.38	−60	6	34			
5	Middle occipital gyrus, area hOc4la (prob. 81%)	7.12	−52	−76	4			
	Middle occipital gyrus	6.66	−36	−72	12			
	Middle temporal gyrus, area hOc5 (V5/MT, prob. 12%)	6.52				42	−66	4
	Middle temporal gyrus	5.85	−48	−62	6			
**Imagination**								
1	Precentral gyrus	5.61	−18	−12	72			
	Superior frontal gyrus*	4.07				16	−8	72
	Supplementary motor area (BA 6)*	4.04				14	−10	66

For simplicity, contrast images were named as *Execution*, *Observation*, and *Imagination*, respectively.

*ROI were defined with reference to the WFU Pickatlas/AAL.

In the *Execution* task, bilateral activation of the Paracentral lobule (Area 4a) was observed; this cluster of activation was very large and included part of bilateral primary sensory (post central gyri) and motor areas (pre central gyri), premotor areas, SMAs, secondary somatosensory areas (SII) and left Putamen. Different loci of activity resulted in the right hemisphere of the cerebellum (H IV—H V), and Vermis.

In the *Observation* task, activations in the left Putamen, bilateral Thalamus, right mid-cingulate cortex (MCC), right precentral, right middle frontal, left precentral gyrus (area 44), left inferior parietal lobule and left superior parietal lobule (area 5L, SPL), left middle temporal, right middle temporal (area hOc5, V5/MT), left middle occipital and middle temporal gyri (area hOc4la) were found, whereas in the *Imagination* task, right SMA (BA 6), right superior frontal, and left precentral gyrus were observed.

**Figure 2:**
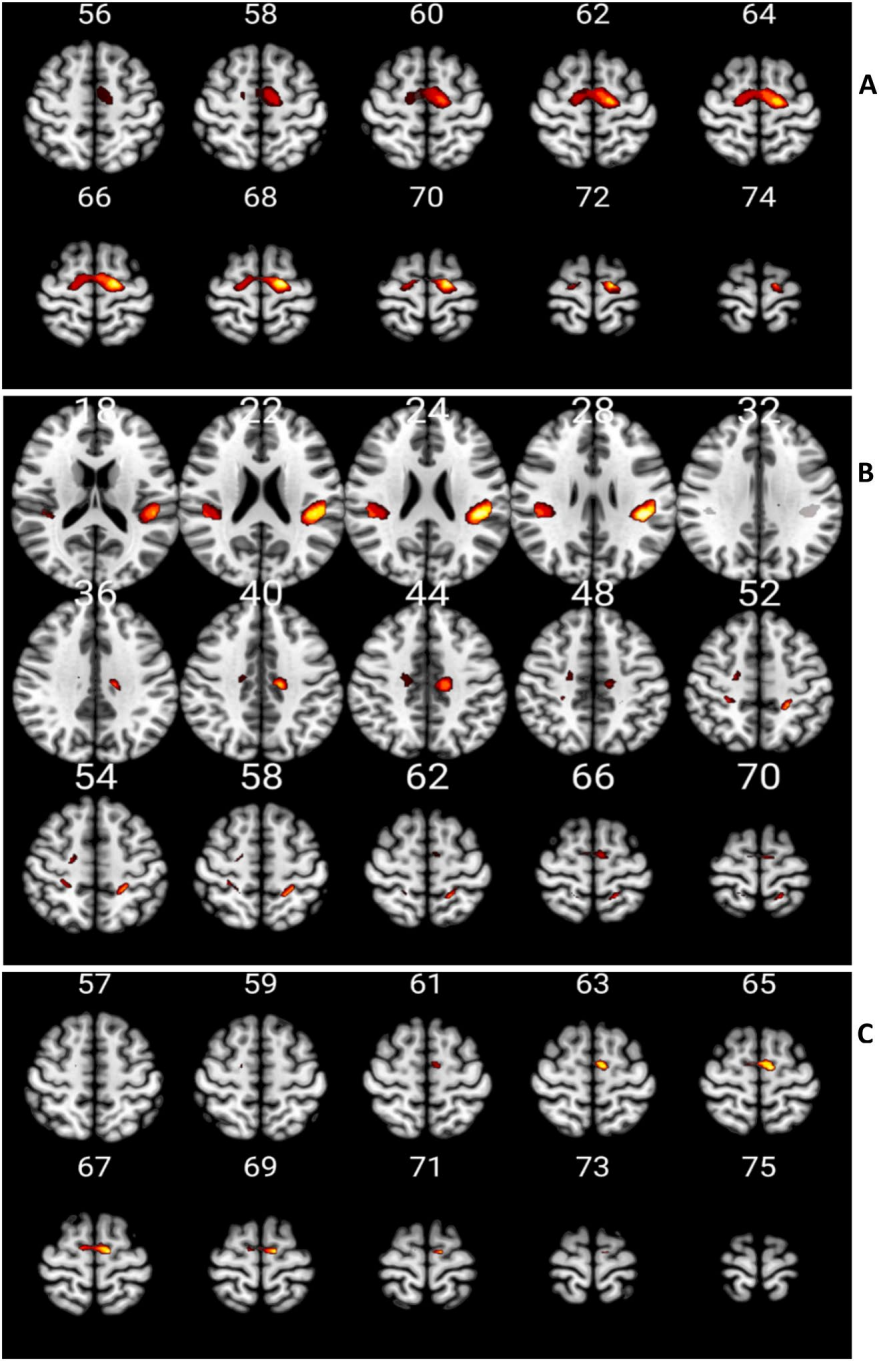
Results of conjunction analysis. The statistical parametric maps show areas that resulted to be activated by both *Execution* ∩ *Observation* **(A)**, *Execution* ∩ *Imagination* **(B)** and *Execution* ∩ *Observation* ∩ *Imagination* **(C)** task conditions. The list of these areas is provided in Table 2. Clusters determined by Z > 3.1, cluster significance threshold of P < 0.05 corrected^38^.

According to conjunction analyses, *Execution* ∩ *Observation* resulted in common activations in the right postcentral gyrus (area 5L, SPL; area 3b), bilateral posterior-medial frontal gyrus, right mid-cingulate cortex (MCC), left superior parietal lobule and postcentral gyrus (area 5L, SPL), right supramarginal gyrus (area PFcm, IPL, OP 1), left inferior parietal lobule (area OP 1) (Fig. 2A). *Execution* ∩ *Imagination* showed activations in SMA (Fig. 2B). Finally, *Execution* ∩ *Observation* ∩ *Imagination* revealed common activations in a small area of right Superior Frontal Gyrus (SFG) and bilateral SMA (Fig. 2C) (Table 2).

**Table 2:** MNI standard brain coordinates of the local maxima of BOLD-signal increases as revealed by the three conjunction analyses.

Cluster index	Anatomical region	Z	Left (mm)			Right (mm)		
Observation ∩ Execution			x	y	x	x	y	x
1	Postcentral gyrus, area 3b (prob. 35%), area 4p (prob. 23%), area 2 (10%)	4.29				28	−36	56
	Postcentral gyrus, area 4p (prob. 17%), area 3a (15%)	3.97				26	−34	48
	Postcentral gyrus, area 5L (SPL, prob. 34%), area 5 M (SPL, 26%), area 3b (26%)	3.74				12	−46	72
2	Posterior-medial frontal gyrus: supplementary motor area (BA 6)*	3.83	−8	−10	68			
	Posterior-medial frontal gyrus: supplementary motor area (BA 6)*	3.38				6	−8	68
3	Middle cingulum (BA 24)*	3.45				16	−20	42
4	Postcentral gyrus, area 5L (SPL, prob. 59%), area 2 (prob. 42%), area 4p (6%)	5.32	−24	−42	56			
	Superior parietal lobule, area 5L (SPL, prob. 68%), area 5 M (SPL, 12%), area 4a (12%)	4.26	−18	−48	72			
5	Cingulate gyrus*	4.83	−14	−22	40			
6	Supramarginal gyrus, area Pfcm (IPL, 45%), area OP1(SII, 20%)	4.57				48	−34	24
7	Inferior parietal lobule	5.8	−42	−32	26			
**Imagination ∩ Execution**								
1	Right superior frontal gyrus: supplementary motor area*	4.04				14	−10	66
**Observation ∩ Imagination ∩ Execution**								
1	Superior frontal gyrus*	3.46				16	−10	60
2	Supplementary motor area*	4.77	−12	−12	66			
	Supplementary motor area*	3.97				12	−8	66

Indeed, common activation areas for walking *Observation* and *Execution*, *Imagination* and *Execution*, and *Observation*, *Imagination* and, *Execution* are displayed.

*ROI were defined with reference to the WFU Pickatlas/AAL.

### Physiological data results

#### RR

**Figure 3:**
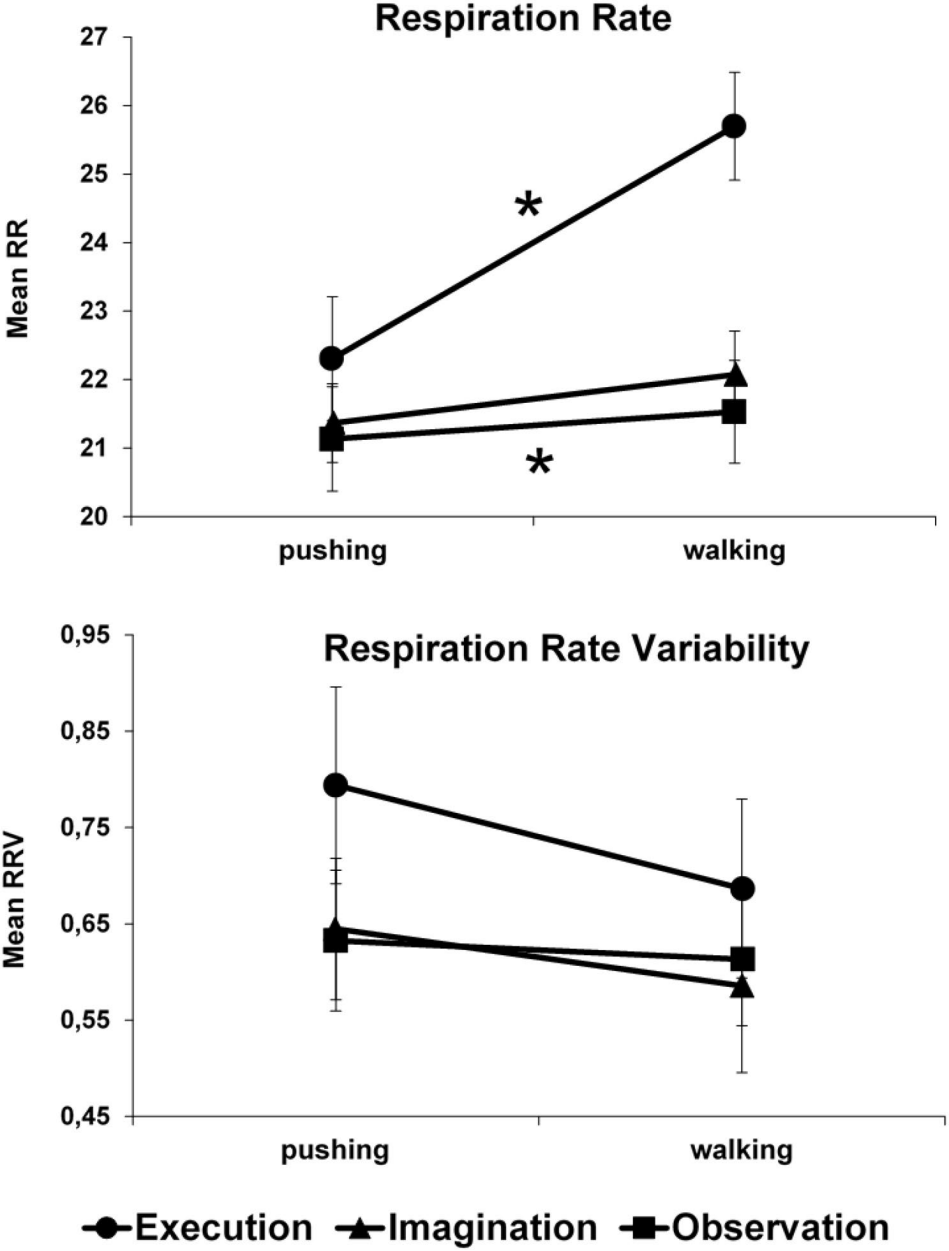
Effect of execution, observation, and imagination of the walking action compared to the pushing action on the two physiological measures: respiration rate, and respiration rate variability. Asterisks indicate significant effects.

The main effect of *Condition* (Execution vs. Observation vs. Imagination) was significant, *F*(2, 28) = 12.331, *p* < 0.001, η^2^_p_ = 0.47. Paired-samples *t*-tests showed that respiration rate was higher in the *Execution* condition (24 BPM) relative to the *Observation* condition (21.3 BPM), *t*(14) = 4.441, *p* < 0.001, *d*_*z*_ = 1.15 and the *Imagination* condition (21.7 BPM), *t*(14) = 3.306, *p* = 0.005, *d*_*z*_ = 0.85 whereas no difference resulted between *Observation* and *Imagination* conditions, *t*(14) = 0.927, *p* = 0.369, *d*_*z*_ = 0.24 (Bonferroni-corrected *p* level = 0.016). The main effect of *Task* (Walking vs. Pushing) was significant, *F*(1, 14) = 28.057, *p* < 0.001, η^2^_p_ = 0.67 (Pushing = 21.6 BPM; Walking = 23.1 BPM). The *Condition* x *Task* interaction was significant, *F*(2, 28) = 21.345, *p* < 0.001, η^2^_p_ = 0.60. Respiration rate increased significantly in the walking task relative to the pushing task in the *Execution* condition (25.7 vs. 22.3 BPM), *t*(14) = 5.631, *p* < 0.001, *d*_*z*_ = 1.45, in the *Observation* condition (21.5 vs. 21.1 BPM), *t*(14) = 2.995, *p* = 0.010, *d*_*z*_ = 0.77, and tended to increase in the *Imagination* condition (22.1 vs. 21.4 BPM), *t*(14) = 2.096, *p* = 0.055, *d*_*z*_ = 0.54 (Bonferroni-corrected *p* level = 0.016) (Fig. 3, upper panel). To determine whether participants’ ability to imagine correlated with the physiological response to imagined walking and pushing actions, Pearson correlation coefficients were used (alpha level = 0.01, two-tailed), which assessed the relationship between KVIQ-V and KVIQ-K scores, and the effect of task in the imagination condition (i.e., the difference between walking and pushing task). Correlations were not statistically significant between KVIQ-V scores and the task effect, *r*(13) = -0.22, p = 0.426, and between KVIQ-K scores and task effect, *r*(13) = -0.12, p = 0.663.

#### RRV

The main effect of *Condition* was not significant, *F*(2, 28) = 1.402, *p* = 0.263, η^2^_p_ = 0.09. The main effect of *Task* was significant, *F*(1, 14) = 7.276, *p* = 0.017, η^2^_p_ = 0.34, displaying a decrease of respiration rate variability in the more demanding Walking task (RMSSD = 0.628 relative to the less demanding Pushing task (RMSSD = 0.690). The nonsignificant interaction between *Condition* and *Task*, *F*(2, 28) = 1.302, *p* = 0.288, η^2^_p_ = 0.08, indicated a similar decreasing pattern in the three *Execution*, *Observation* and *Imagination* conditions (see Fig. 3, lower panel). Correlations between KVIQ-V scores and the task effect, and between KVIQ-K scores and task effect were not statistically significant: *r*(13) = -0.26, p = 0.347, and *r*(13) = -0.13, p = 0.638.

In sum, we observed overall higher RR when actual movements were carried on, relative to when the same participants rested on the bed while watching a video clip of someone walking/pushing, or imaging themselves walking/pushing. In the *Execution* condition, RR resulted higher in the walking task relative to the pushing task, as a consequence of the mental representation of walking action. Crucially, although attenuated relative to actual execution^37^, the RR responses also increased when the participants (while resting on the scanner bed) observed someone walking relative to when they observed the less demanding pushing action. The same pattern was numerically observed when participants imagined themselves walking versus pushing (Fig. 3, upper panel). This last result did not correlate with the participants’ ability to imagine; this suggests that the lack of a significant difference between walking and pushing actions was not related to eventual insufficient ability to perform the imagination task. An overall decreasing RRV was observed when participants were engaged in the walking movement relative to the pushing movement, with a similar decreasing slope in the three *Execution*, *Observation*, and *Imagination* conditions (Fig. 3, lower panel).

#### GLM analysis of physiological data and fMRI results

Given that the only RR exhibited significant changes between walking and pushing tasks, we have run further GLM analysis only using RR parameters as regressors. The analysis was conducted separately for each condition.

**Figure 4:**
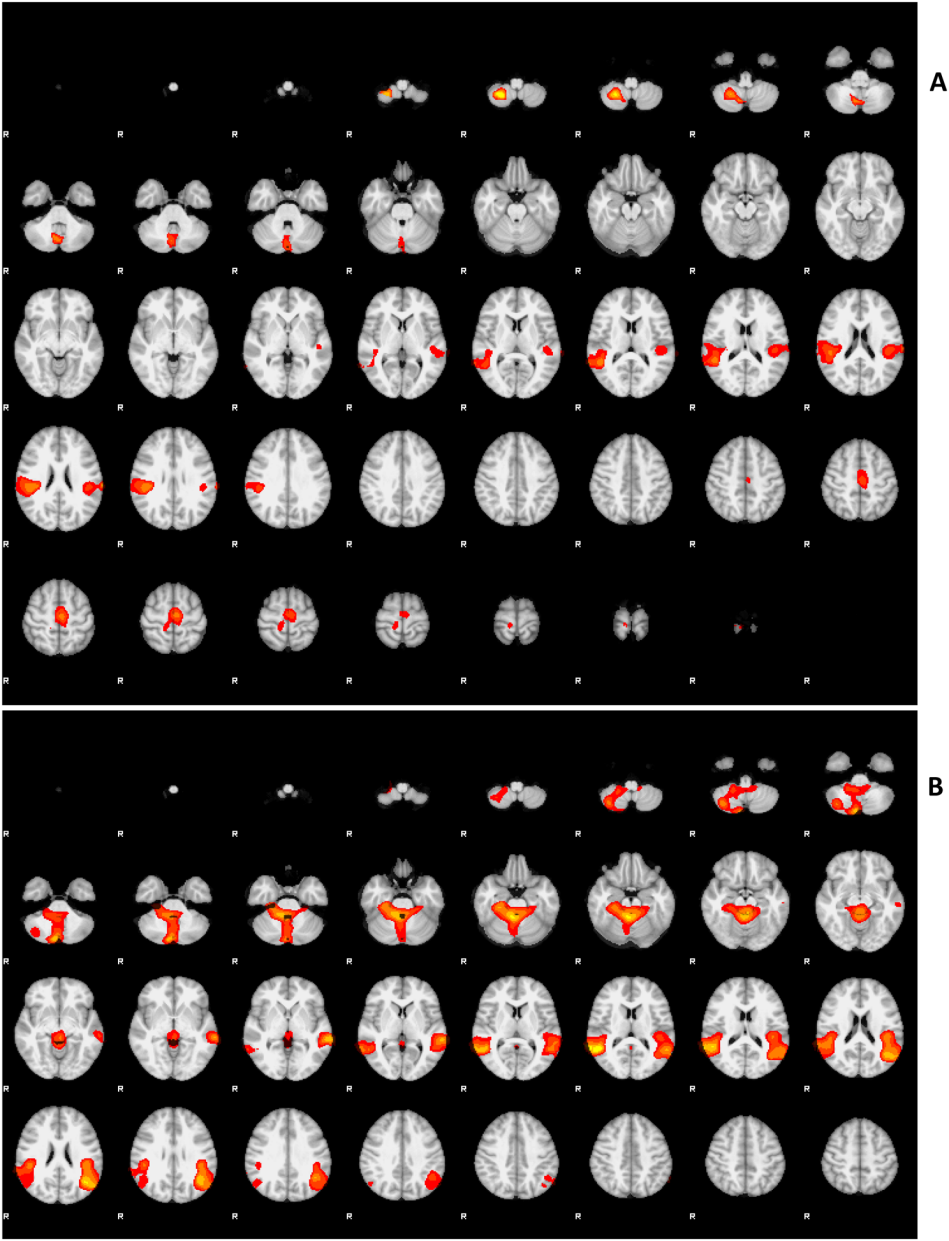
GLM analysis showing the effect of the RR time course during *Execution* condition **(A)** and all three conditions (*Execution*, *Imagination,* and *Observation*) **(B)** in the group. The activity displayed in this parametric map was explained by the time course of RR parameters not using estimation parameters of the tasks performed by the subjects. Clusters determined by Z > 3.1, cluster significance threshold of P < 0.05 corrected^38^.

During the *Execution* condition we have found significant results (cluster significance threshold of *p* < 0.05 ^38^; group analysis revealed that the activity of four large areas was explained by the time course of RR parameters. The first cluster included a portion of right Angular gyrus, right parietal operculum (the area corresponding to right SII) and right middle temporal gyrus; the second cluster is located between left supramarginal gyrus, left parietal operculum (the area corresponding to left SII), left middle and superior temporal gyrus; the third cluster occupies part of the right inferior lateral cerebellum (cerebellum_8 in AAL), caudal (vermis_8 in AAL) and cerebral crus (Cerebelum_Crus2 in AAL); the fourth cluster lies in the right postcentral gyrus, right paracentral lobule and bilateral supplementary motor area (Fig. 4A).

When *Execution* was analyzed together with *Observation* and *Imagination* we found significant activations that included different areas compared to the ones obtained in the *Execution* condition alone. In this case, results showed 3 large clusters: the first cluster occupied the vermis of cerebellum (Vermis_1_2 and Vermis_3 in AAL), right cerebellum (Cerebelum_3 and Cerebelum_7b in AAL) and the dorsal part of the brain stem including medulla oblongata, pons, and midbrain; the second cluster was a large area distributed between left angular gyrus, left supramarginal gyrus, left middle temporal gyrus and left parietal operculum (the area corresponding to right SII); finally, the third cluster was found in the right angular gyrus, right middle and superior temporal gyri and right parietal operculum (the area corresponding to right SII) (Fig. 4B). Neither *Observation* nor *Imagination* yielded significant correlation alone.

## Discussion

The present study provided a unified fMRI and physiological investigation of the Mirror Neuron system for walking actions.

We demonstrated first unified evidence for a common MNS activation in walking execution, observation, and imagination. Crucially, we outlined the existence of a close link between MNS and autonomous nervous system and identified brain areas that are the most likely candidates for the modulation of ANS response during MNS activity.

Basically, we observed brain activity in areas specific for the kind of performed tasks: walking *Execution*, *Observation,* and *Imagination*. These results were in line with previous studies; thus confirming the validity of our paradigms and general set up. In addition to task-specific areas, we observed areas which were activated during different task conditions. During both *Execution* and *Observation,* several frontoparietal areas resulted activated (Fig. 2A); interestingly we found that the inferior parietal lobule was activated in both the task conditions, confirming the importance of this area in action representation^39^. Furthermore, the final conjunction analysis revealed bilateral activation of SMA shared by all three task conditions (Fig. 2C). These results support the view that these areas represent the neural underpinning of a common motor representation for walking. In particular, bilateral SMA would subserve all three motor-cognitive processes of *Execution*, *Observation,* and *Imagination*, thus suggesting it is the core part of a mirror neuron system for lower limbs actions.

Physiological results for RR (and RRV) measures support the claim that a vegetative response is also produced in observed and imagined motor activities beyond the performed ones, and that this response is proportional to the intensity of such perceived and imagined actions. Specifically, increased RR in the walking task relative to the baseline task (pushing) was expected and may be expression of the locomotion respiratory coupling (LRC), a mechanism adapting ventilation to changes in metabolic requests during locomotion^40^. In keeping with this, there is evidence that SMA is involved in respiratory actions^41,42^. The relevant evidence for the aim of the present study is that also during the *Observation* and (tendentially) the *Imagination* conditions the same mechanism seems to be operating, thus indicating that the mere mental representation of such motor action can induce changes in the ANS response. To note, LRC may be in turn affected by such motor resonance processes; possibly involving the contribution of central pattern generators (CPGs)^40^. This represents, in our view, a clear behavioral evidence of a functional connection between the MNS and the ANS. Indeed, the ANS responses would offer one further level of evidence that the cognitive processes activated in actual movement execution are involved to the same extent in movement observation and probably also in movement imagination^1^.

**Figure 5:**
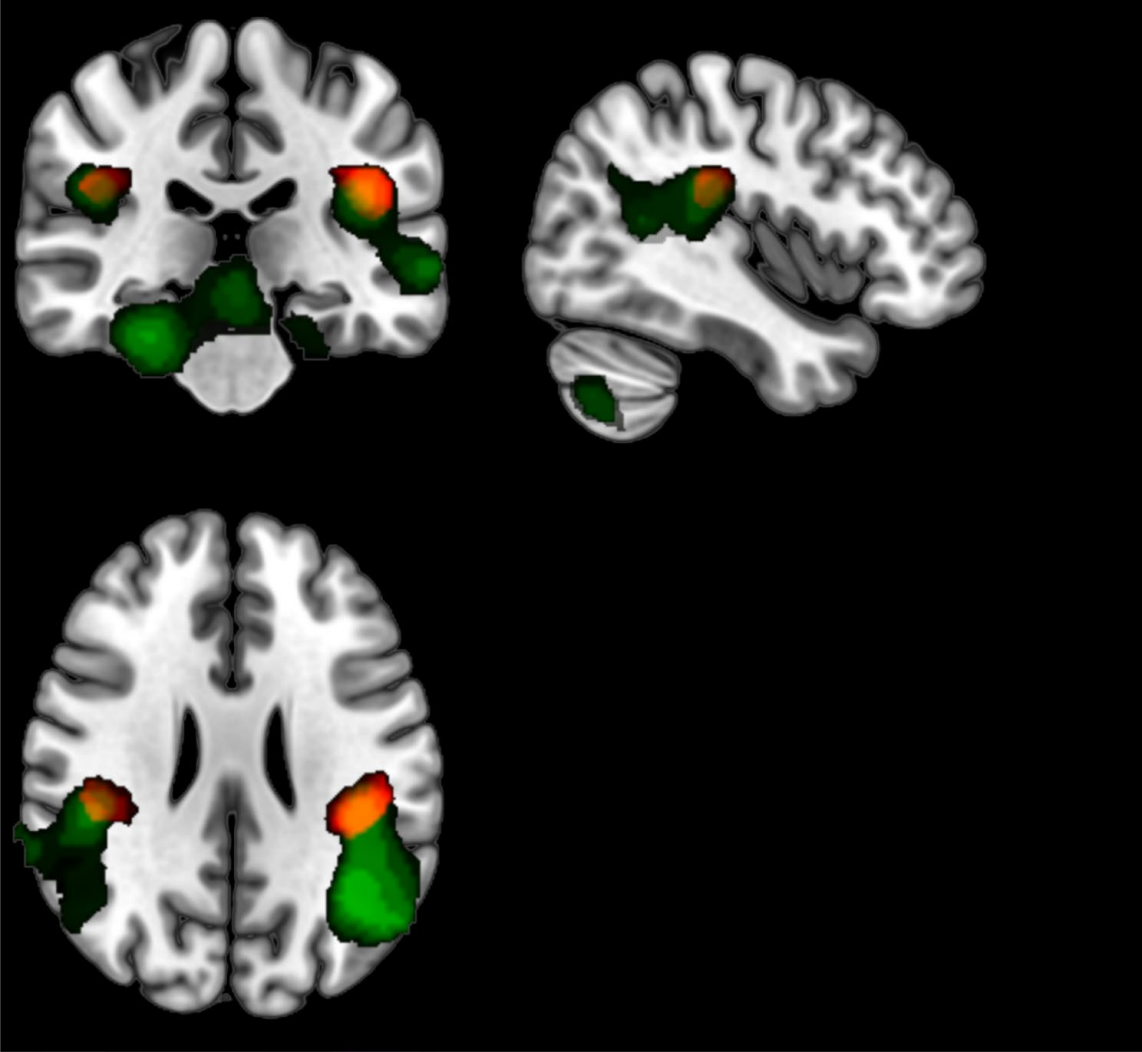
The green areas show the result of the conjunction analysis *Execution* ∩ *Observation,* areas that resulted to be activated by both *Execution* and *Observation* conditions. The red areas show the result of GLM analysis using RR parameters as regressors during all three conditions (*Execution*, *Imagination,* and *Observation*). The yellow areas are the common areas that correspond with left and right parietal opercula, OP1 (SII).

To address the hypothetical functional link between the mental representation of walking and the modulation of ANS responses, we combined the RR physiological parameter and the fMRI data into an ad-hoc GLM design. To note, in this analysis no categorical regressor was used to model the walking/pushing tasks, but only the RR variation across time. During walking *Execution*, brain activities were highly compatible with activation patterns typical for a motor task: right parietal operculum (SII), left parietal operculum (SII), right inferior lateral cerebellum, caudal and cerebral crus, right postcentral gyrus, right paracentral lobule, and bilateral SMA; in addition to some other areas: right Angular gyrus, right middle temporal gyrus, left supramarginal gyrus, left middle and superior temporal gyrus. The effect of RR in the brain during all three conditions taken together also produced highly significant results but the scenario was slightly different. In this case, three large clusters resulted activated by the RR parameter (Fig. 4B). The first cluster included large portions of the cerebellum (vermis, right cerebellum) and the dorsal part of the brain stem including medulla oblongata, pons, and midbrain; these parts of the brainstem play important roles in the regulation of respiratory function, helping to control breathing rate. The dorsal part of the brainstem which was found activated in the response to the RR parameter includes the dorsal respiratory group, which has the most fundamental role in the control of respiration and maintains the rate of respiration^43,44^. Additionally, it was suggested that the Midbrain Periaqueductal Gray, which is also partially included in the activated cluster, “serves as the behavioral modulator of breathing”^45^. The other two clusters occupied bilateral parts of the brain; on the left, the angular gyrus, supramarginal gyrus, middle temporal gyrus, and parietal operculum were activated while on the right, angular gyrus, middle and superior temporal gyri, and parietal operculum were parts of the controlateral cluster. The activation of bilateral parietal opercula is remarkable because these areas were already found activated by both the *Execution* and *Observation* conditions (Fig. 5); the coordinates of these loci correspond to the anatomical site of SII: the parietal operculum (OP 1 according to Eickhoff et al., 2006)^46^. Electrophysiological studies in monkeys have provided evidence that SII contains distinct, bilateral body representations^47,48^. In humans it consists of four different sectors (OP1-OP4) with multiple representations of the body. It is strictly connected with SI, premotor cortex and Broca’s region^46,49^. Brain imaging studies have shown its involvement in sensorimotor integration^50,51^ and during tactile stimulation ^52–55^ and tactile object recognition^56^. SII has been also involved in tasks requiring attention to interoceptive stimuli and internal awareness^57^. Sensory afferents to SII coming from the respiratory system have been reported in lower species^58^. Given this background, SII would be involved in the generation of somatosensory internal (kinaesthetic) representations by capturing information from the external world; then it would be also responsible for the modulation of the appropriate autonomous response, more precisely respiration rate changes.

Our study provided some indications that the activity of bilateral SII would be related to the changes of RR not only during walking *Execution* but during walking *Imagination* and *Observation* as well. It has been already suggested that the MNS is involved in action recognition^59^, at least when an observed action is part of the observer’s motor repertoire^60,61^. The results of the present study allowed us to cautiously extend the range of this notion, suggesting that during action observation (and tendentially action imagination) not only the visual and kinematic aspects of an action, but also its autonomic correlates are re-enacted: RR in the present study. Such combined mechanisms would be crucial to explain how, for instance, an observed action is also complemented by the understanding of the efforts and the fatigue behind it; thus allowing to discriminate between heavier and lighter workloads. We suggest that the most likely candidate area to play a crucial role in re-enacting the autonomic functions related to a given action could be the SII. Thus from our perspective, to combine a specific motor or sensorial experience with its autonomic correlates would be a general function of SII.

In conclusion, the present study has revealed dorsal premotor cortex and SMA as crucial areas for walking, when executed, observed or imagined. Even more interestingly, it has shown that, while re-enacting actions, individuals also re-enact their autonomic correlates. SII would candidate to resonate with other individuals’ autonomic functions during action observation.

## Materials and methods

### Participants

Twenty-two healthy participants (mean age 25.36 years, SD 4.79) of which eight females were recruited; all subjects were right-handed as determined by the Edinburgh Handedness Inventory^62^ (mean LQ =  + 87.8; SD = 15.5; range = 55.6–100) (see supplementary information for further detail*s*). Experimental procedures were approved by the Ethics Committee of the Medical Faculty of the RWTH Aachen University, and performed in accordance with its guidelines and regulations. All participants gave written informed consent to participation and received compensatory payment.

### Design

The experiment was conducted on a 3 T Siemens Prisma (Erlangen, Germany) with 20-channel head coil. Participants laid on the scanner bed while having a foam pillow under their legs/knees. An MRI compatible frame with a plastic rolling cylinder was fixed on the MR bed (outside the scanner). Participants were able to move their legs to roll the cylinder and reproduce a walking-like movement. To minimize trunk and head movements, head and waist were immobilized with foam pads and 2 belts, respectively. Visual stimuli were projected through an MR-compatible monitor placed behind the scanner and seen by the participant by mean of a mirror integrated within the head coil. Stimulus display was controlled by the Presentation software (Neurobehavioral Systems, http://www.neurobs.com) triggered by the scanner. During the fMRI acquisitions, the respiratory signal was collected with the BIOPAC MP150 hardware interfaced with the MR scanner. The respiratory signal was recorded using the TSD221-MRI transducer (https://www.biopac.com).

### Procedure and experimental conditions

Before the participant entered the scanner, the printed text of the general instructions was given to her/him. Participants were tested in one session divided into three tasks: execution, observation, and imagination. Each task was performed in one block of trials and repeated three times for a total of nine blocks in a pseudorandomized order. Each block consisted of 45 trials: 22 experimental trials alternated with 23 baseline trials (i.e., first and last trials were baselines) (Fig. 1). In the *execution* condition-*experimental* trials the participant was instructed to execute a walking movement, whereas in the *execution* condition-*baseline* trials she/he had to gently push the cylinder with both feet. These baseline trials were thought to activate the same neuromuscular pattern involved in the walking task but not the higher-level neural pattern specific for walking action.

During the planning and the implementation of the experimental procedure, we aimed at allowing the participant to perform her/his walking movement so that it resembled as much as possible a regular walking. To obtain this, we shaped an adjustable semi-hard foam “pillow” that was meant to support the legs when in rest position, and the glutei during the walking movement. In this last condition, the pillow left the legs free to perform the walking movement on the rolling cylinder while leaving the pelvis partly free to move. Indeed, the pillow pressed against the glutei hard enough to stabilize the trunk and minimize head movements on the one hand, and support the leg movements that had no constrain on the knees and ankles, on the other hand. This allowed the participant to reproduce a movement very similar to walking by placing alternatively the heels on the cylinder and pushing down just enough to slide each foot along the entire length of the sole.

During this execution condition, the participants kept their eyes at a fixation cross displayed on the monitor. In the *observation* condition-*experimental* trials, the same participant was instructed not to move and to watch a short video of a female or male actor walking on the same rolling cylinder. The video focused on legs and feet taken from randomly alternated frontal (i.e., egocentric perspective, as if one filmed her/himself walking), left, and right views (see supporting materials). The corresponding *baseline* was to watch a set of pictures depicting someone pushing the cylinder with her/his feet from the same three perspectives. Each video clip lasted 18 s and was divided into three 6-s portions, one for each view. In the *imagination* condition-*experimental* trials the participant was instructed not to move, and to imagine (with closed eyes) her/himself walking on the cylinder, whereas in the *baseline* trials she/he will imagine her/himself to push it. Each trial started with 2 s recorded audio instructions (e.g. in the execution condition a male or female voice said “laufen” or “drucken” to indicate participants to walk on or push the cylinder, respectively), followed by 18 s in which participants had to perform the instructions. Therefore, each block condition lasted 15 min (7 min experimental and 8 min baseline trials); their order was randomized between participants. Short resting pauses (1 min, approximately) were given in between. Three functional runs were acquired (one for each block condition), followed by one structural run (duration: 5 min). The total scanning session lasted approximately 60 min.

### MRI data acquisition and analysis

Denoised data (see supplementary information for pre-processing of fMRI data) were analyzed using GLM approach with FSL-FEAT v. 6.00 (FMRIB's Software Library). Head motion parameters, as estimated by MCFLIRT motion correction in the Pre-processing, were included as confound in the model. The three tasks (walking, walking imagination and walking observation) were analyzed separately; any of them represented the predictor of interest and was compared to its own baseline as described in the stimuli paragraph. To account for the hemodynamic delay, the boxcar waveform representing the baseline and task conditions was convolved with an empirically founded double-Gamma hemodynamic response function. Subjects performed three pseudorandomized repetitions of every task; during a second-level analysis, these repetitions were concatenated for any subject assuming equal variance by using a fixed-affect analysis. As a result of this process, we obtained activation maps for any subject displaying the contrast activations for the three tasks: *walking execution* vs *pushing execution*, *walking observation* vs *pushing observation,* and *walking imagination* vs *pushing imagination.* In addition, to search for activated areas showing within and between-group effects, three voxel-wise random effect group analyses (third level) were performed as well with FSL. Activated areas were obtained from the group activation maps of the whole brain analysis considering those voxels showing a significant response (P < 0.05 FWE corrected) to any experimental condition. Finally, the three random effect group analyses were compared using a conjunction analysis to investigate the areas of the brain that responded to (i) execution and observation, (ii) execution and imagination, (iii) execution, observation, and imagination tasks.

### Physiological data acquisition and analysis

As for indicators of ANS activity we have acquired respiratory signals from the participants; the acquisition of the signals was synchronized with tasks and fMRI images. Of the physiological indexes, respiration rate (and its changes) is the only index that is directly observable; being therefore integral part of an observed motor effort. Incomplete data have been excluded from analyses (see supplementary information for pre-processing of physiological data). This resulted in data signals from fifteen participants (mean age 26.53 years, SD 4.85, six females) that underwent further analysis. For these participants, times of data acquisition ranged between 11:30 AM to 4:30 PM, all of them were non-smokers, used to have moderate physical ativity (about 2 h a week), and were required not to consume foods containing caffeine within at least 3 h of the start of the measurement. Their body mass index (BMI) averaged 23.92 kg/m^2^ (sd = 3.89). Two parameters were computed: respiration rate (RR) and respiration rate variability (RRV). Mean RR and RRV were submitted to two separate repeated-measures analyses of variance (ANOVAs) with *Condition* (execution vs. observation vs. imagination), and *Task* (pushing vs. walking) as within-participants factors. Values of *p* < 0.05 were considered statistically significant. Paired-sample *t*-tests were performed as post-hoc comparisons with Bonferroni corrected *p* values. All statistical tests were performed in SPSS (IBM, USA). An open-source tool was used to compute Cohen’s *d*_*z*_ effect size for the *t*-tests (https://webpower.psychstat.org/models/means01/effectsize.php).

### GLM analysis of physiological data and fMRI

Finally, estimation parameters of the physiological data were calculated using separate regressors for RR and RRV parameters. In order to spotlight brain areas that could be responsible for changes in the ANS response, as measured by the two acquired parameters, we combined these parameters and fMRI data in a GLM approach. The physiological data were acquired during all fMRI sessions; the acquisition of different signals was synchronized in order to compare different time courses. For this purpose denoised data were analyzed using GLM approach (Friston et al.^63^ ) with FEAT with identical set-up described above, but in this case, only the estimation parameters of the physiological data were used as predictor of interest instead of the tasks performed by the subjects (walking, walking-imagination and walking-observation). The analyses were performed (1) separately for the physiological parameters and (2) separately for fMRI sessions acquired during different task conditions, (3) but the task was not introduced in this GLM-analysis as regressor to see only the effect of physiological data.

## Supplementary Information

Supplementary Video 1.

Supplementary Information.

## Data Availability

The datasets generated during and/or analysed during the current study are available from the corresponding author on reasonable request.
