# Supplementary material for: Respiratory function modulated during execution, observation, and imagination of walking via SII
Source: Sci Rep. 2021 Dec 9;11:23752. doi: 10.1038/s41598-021-03147-5 (PMC8660877; doi:10.1038/s41598-021-03147-5)
Supplement: Supplementary file 2 — Supplementary Information. [file 41598_2021_3147_MOESM2_ESM.docx]

**Supplementary information**

**Respiratory function modulated during execution, observation, and imagination of walking via SII**

**Materials and Methods**

**Participants.** The following exclusion criteria were applied: neurological disease (e.g., history of a craniocerebral trauma), dementia or cognitive impairment, physical illness and mental or behavioral disorder potentially interfering with the experimental investigation, seizure disorders, operations on the heart and/or brain, drugs assumption, alcohol abuse, electromagnetically modifiable or metal medical devices, metal parts in the body, pregnancy. To assess the ability of participants to engage in motor imagery, we used the short version of the Kinesthetic and Visual Imagery Questionnaire (KVIQ-10)^1^. Mean group score for the visual imagery subscale (KVIQ-V, presented first) was 32.02 (SD = 7.8), and for the kinesthetic imagery subscale (KVIQ-K) was 32.53 (SD = 8.2).

**MRI Data acquisition and analysis.** We acquired 9 series of 153 T2*-weighted whole-brain volumes using a gradient-echo EPI sequence (TR 2000 ms, TE 38 ms, flip angle 77°, 34 contiguous axial slices with 4 mm thickness, no gap, matrix 64×64, in-plane resolution of 4×4 mm). In addition, we acquired a high-resolution structural scan for co-registration using a 3D MPRAGE sequence with 208 contiguous sagittal slices of 1 mm thickness (TR 2400 ms, TE 2.18 ms, echo time 8.9 ms, flip angle 8°, matrix size 224×224, isotropic voxel dimensions of 1×1×1 mm). Both functional and structural images series underwent a quality assurance protocol, including visual inspection; none of the participants’ data showed artefacts.

Data pre-processing was carried out using FEAT (FMRI Expert Analysis Tool) Version 6.00, part of FSL (FMRIB's Software Library). We first discarded the first 13 images consisting of 3 preparation volumes and 1 complete baseline block (10 volumes) of each functional series to avoid T1 saturation effects; the remaining 140 volumes were utilized. Registration to high resolution structural and standard space images was carried out using FLIRT^2,3^. Registration from high resolution structural to standard space was then further refined using FNIRT nonlinear registration^4,5^. The following pre-statistics processing was applied; motion correction using MCFLIRT^2^; slice-timing correction using Fourier-space time-series phase-shifting; non-brain removal using BET^6^; spatial smoothing using a Gaussian kernel of FWHM 12.0 mm; the FWHM value was decided relatively big to reduce the effect of head movement produced by subjects during the walking task. Finally, the entire 4D dataset was grand-mean intensity normalized by a single multiplicative factor. Motion estimation parameters were evaluated and those sessions that exhibited absolute movements exceeding the range of ± 3 mm were discarded.

In order to reduce the impact of movement during the walking tasks on fMRI analysis, all 4D datasets were decomposed by mean of Independent Component Analysis (ICA) technique, and components related to movement artifacts were removed. For this purpose, we have used *ICA-AROMA* (ICA-based Automatic Removal Of Motion Artifacts)^7^ a data-driven method to identify and remove automatically motion-related independent components. Preprocessed fMRI data were decomposed with ICA-AROMA into a set of independent components; then ICA-AROMA automatically identifies which of these components are related to head motion, by using four robust and standardized features^8^. The identified components are then removed from the data through linear regression as implemented in fsl_regfilt. The *aggressive* denoising options of ICA-AROMA have been implemented in this analysis.

In the end, fMRI denoised data were analyzed using the GLM approach^9^ with FEAT. Time-series statistical analysis was carried out using FILM prewhitening to make the statistics valid and maximally efficient with local autocorrelation correction^10^.

The anatomical locations of activation patterns were identified with reference to the Jülich cytoarchitectonic probabilistic maps of the human brain, using the SPM anatomy toolbox version 2.2b. The WFU Pickatlas/AAL^11^ was used to identify brain areas that were not indexed in the Jülich maps.

**Physiological data acquisition and analysis.** The respiration signal was recorded at the acquisition frequency of 2000 Hz, pre-processed with Acq*K*nowledge 5.0, and analyzed with MATLAB R2017b. For each participant, the signal was first down-sampled to 100 Hz for faster computation and separated into corresponding epochs of execution, observation, and imagination, resulting in a total of 9 epochs based on the experimental design. Once separated, the correct portion of the signal corresponding to the scans was selected through the recorded dummy scans and trigger signals. The respiratory signal was bandpass filtered (0.05 Hz – 1 Hz, finite impulse response (FIR) filter, number of coefficients determined by 4 x (sample rate divided by lowest cut off frequency)) based on its power spectral evaluations. Signal segments with large artefacts were discarded and those with small artefacts were mathematically transformed or set to 0.

Two parameters were computed: respiration rate (RR) and respiration rate variability (RRV). The RR was calculated by counting the number of peaks within each segment of task and baseline for each epoch, per minute (breaths per minute - BPM). The RRV was obtained by calculating the Root Mean Square of Successive Differences (RMSSD) and by ensuring that only complete cycles were used within each epoch. The RMSSD is the root mean square of successive differences between normal peaks of breaths. This value is obtained by first calculating each successive time difference between breaths in seconds. Each of the values is then squared and the result is averaged before the square root of the total is obtained^12^. RRV may provide important information about respiratory regulation; it was associated with age and illness; the decrease of variability is correlated with a decrease of adaptability or increase of stress in several clinical situations^13,14,15,16^. RRV was found reduced during sleep, being greater in wakefulness than in any sleep stage. Finally, it was found a reduction of RRV during sustained attention tasks^17^.

**GLM analysis of physiological data and fMRI**. The regressors for RR and RRV parameters were calculated as follows: the preprocessed physiological data was analyzed to determine RR for the whole experiment. One value for each image volume was calculated by the true corresponding value. The RRV was calculated for both baseline and task separately, resulting in one value per block. This was then replicated ten times since each block consisted of ten volumes.

**Video S1.** A sample video clip of an actor walking on the rolling cylinder, provided in the observation condition-experimental trials.

**References**

1. Malouin, F. *et al.* The kinesthetic and visual imagery questionnaire (KVIQ) for assessing motor imagery in persons with physical disabilities: A reliability and construct validity study. *J. Neurol. Phys. Ther.* **31**, 20–29 (2007).

2. Jenkinson, M., Bannister, P., Brady, M. & Smith, S. Improved Optimization for the Robust and Accurate Linear Registration and Motion Correction of Brain Images. *Neuroimage* **17**, 825–841 (2002).

3. Jenkinson, M. & Smith, S. A global optimisation method for robust affine registration of brain images. *Med. Image Anal.* **5**, 143–156 (2001).

4. Anderson, J. L. R., Jenkinson, M., & Smith, S. M. . *Non-linear optimisation. FMRIB technical report*. (2007).

5. Anderson, J. L. R., Jenkinson, M., & Smith, S. M. . Non-linear registration, aka spatial normalisation. FMRIB technical report TR07JA2. (2007).

6. Smith, S. M. Fast robust automated brain extraction. *Hum. Brain Mapp.* **17**, 143–155 (2002).

7. Pruim, R. H. R. *et al.* ICA-AROMA: A robust ICA-based strategy for removing motion artifacts from fMRI data. *Neuroimage* **112**, 267–277 (2015).

8. Pruim, R. H. R., Mennes, M., Buitelaar, J. K. & Beckmann, C. F. Evaluation of ICA-AROMA and alternative strategies for motion artifact removal in resting state fMRI. *Neuroimage* **112**, 278–287 (2015).

9. Friston, K. J. *et al.* Statistical parametric maps in functional imaging: A general linear approach. *Hum. Brain Mapp.* **2**, 189–210 (1994).

10. Woolrich, M. W., Ripley, B. D., Brady, M. & Smith, S. M. Temporal Autocorrelation in Univariate Linear Modeling of FMRI Data. *Neuroimage* **14**, 1370–1386 (2001).

11. Tzourio-Mazoyer, N. *et al.* Automated Anatomical Labeling of Activations in SPM Using a Macroscopic Anatomical Parcellation of the MNI MRI Single-Subject Brain. *Neuroimage* **15**, 273–289 (2002).

12. Soni, R. & Muniyandi, M. Breath Rate Variability: A Novel Measure to Study the Meditation Effects. *Int. J. Yoga* **12**, 45 (2019).

13. Brack, T., Jubran, A. & Tobin, M. J. Dyspnea and Decreased Variability of Breathing in Patients with Restrictive Lung Disease. *https://doi.org/10.1164/rccm.2201018* **165**, 1260–1264 (2012).

14. Papaioannou, V. E., Chouvarda, I., Maglaveras, N., Dragoumanis, C. & Pneumatikos, I. Changes of heart and respiratory rate dynamics during weaning from mechanical ventilation: A study of physiologic complexity in surgical critically ill patients. *J. Crit. Care* **26**, 262–272 (2011).

15. Seely, A. J. *et al.* Do heart and respiratory rate variability improve prediction of extubation outcomes in critically ill patients? *Crit. Care 2014 182* **18**, 1–12 (2014).

16. Segal, L. N. *et al.* Evolution of pattern of breathing during a spontaneous breathing trial predicts successful extubation. *Intensive Care Med. 2009 363* **36**, 487–495 (2009).

17. Vlemincx, E., Taelman, J., Peuter, S. De, Diest, I. Van & Bergh, O. Van Den. Sigh rate and respiratory variability during mental load and sustained attention. *Psychophysiology* **48**, 117–120 (2011).
